# Workplace-based prevention and management of knee pain: a systematic review

**DOI:** 10.5271/sjweh.4195

**Published:** 2025-01-01

**Authors:** Sebastian Venge Skovlund, Mark Skovbye Eg Østergaard, Karina GV Seeberg, Charlotte Suetta, Per Aagaard, Lars Louis Andersen, Emil Sundstrup

**Affiliations:** 1 National Research Centre for the Working Environment, Copenhagen, Denmark.; 2 Department of Sports Science and Clinical Biomechanics, University of Southern Denmark, Odense, Denmark.; 3 Department of Health Science and Technology, Aalborg University, Aalborg, Denmark.; 4 Department of Public Health, University of Copenhagen, Copenhagen, Denmark.; 5 Department of Geriatric and Palliative Medicine, Copenhagen University Hospital, Bispebjerg and Frederiksberg, Copenhagen, Denmark.; 6 Department of Clinical Medicine, University of Copenhagen, Copenhagen, Denmark.

**Keywords:** musculoskeletal disease, occupational medicine, rehabilitation workplace, worker

## Abstract

**Objective:**

Knee pain is highly prevalent and disabling among the general and working population. This systematic review explored the effectiveness of workplace-based interventions on knee pain among workers.

**Methods:**

A PICO-guided systematic search was performed in PubMed and Web of Science Core Collection for articles published from 2003 until January 2023. Eligible articles included randomized and non-randomized controlled trials assessing the effect of workplace-based interventions on knee pain among currently employed adult workers. The quality assessment and evidence synthesis adhered to the systematic review approach, which the Institute for Work & Health developed, and was focused on developing practical recommendations for stakeholders.

**Results:**

Of the 13 identified studies, 11 medium- and high-quality studies were entered into the evidence synthesis. Importantly, none of the included studies specifically aimed at reducing of knee pain. Still, among the included studies, a strong level of evidence suggested no benefit of workplace-based physical exercise/activity intervention on knee pain. The level of evidence was deemed too uncertain to guide current policy/practices for ergonomic and multifaceted interventions. No intervention types were associated with negative effects on knee pain.

**Conclusions:**

The current evidence-base pertaining to workplace-based prevention and management of knee pain is insufficient to guide effective preventive workplace practice or policy development. Considering the global prevalence and health impact of knee pain, development and implementation of effective workplace interventions aimed at prevention and management of knee pain is needed.

Knee pain is highly prevalent globally and affects people of all ages and genders, including the working population (1, 2). Knee pain can be non-specific or caused by specific musculoskeletal disorders (3). Knee osteoarthritis (OA) is the most widespread knee pain-inducing clinical condition and ranks among the most prevalent musculoskeletal disorders overall (3, 6), affecting approximately 16% of the global population aged >15 years and ~23% of adults aged >40 years (7). Knee pain caused by osteoarthritis and other conditions can have profound implications: (i) for individuals in terms of reduced quality of life and functional limitations; (ii) for workplaces through absenteeism- and presenteeism-related productivity loss; and (iii) at the societal level due to increased risk of early retirement and vast socioeconomic costs to increased healthcare use and transfer payments (3, 8, 9). In Australia, the total economic costs associated with lost productivity due to knee osteoarthritis have been estimated to exceed 424.4 billion Australian dollars during the working lifespan (defined as age 15–64 years) (10).

Multiple risk factors for non-specific knee pain and knee osteoarthritis have previously been reported (11–15). Cumulative occupational physical workload, high body mass index (BMI), and previous knee injury have previously been reported to account for 27%, 34%, and 37%, respectively, of hospitalizations related to knee OA (15). Similarly, up to one in seven knee OA cases have been estimated to be directly attributable to work-related factors (14). Specifically, high physical work demands is one example of a well-established risk factor for knee pain (13) and knee OA (12). For instance, certain occupations, ie, construction work, and high exposure to specific occupational physical activities, eg, kneeling and squatting, have been associated with an increased risk of knee pain (13) and knee OA (12).

The influential role of the work environment for the susceptibility to knee pain suggests that work modifications/interventions plausibly could contribute to its prevention and management. Emerging evidence suggests the workplace as a promising arena for prevention and health promotion in general, and in particular for musculoskeletal disorders (16). Owing to the high and rising prevalence of knee OA in the workforce, both leading work environment researchers and the European Alliance of Associations for Rheumatology (EULAR), have recently emphasized the eminent need for increased focus on workplace-based prevention and management of knee OA (1, 17). The issue of occupational knee pain is likely to become more prevalent in the future due to several factors. First, as most European countries are increasing the retirement age, a larger proportion of workers will be experiencing knee pain or knee OA while still in the workforce, potentially having significant consequences for employees and workplaces. Additionally, there is a concerning trend of younger individuals being affected by knee OA, which is often attributed to, eg, increased prevalence of obesity (18). These combined trends suggest that occupational knee pain will be an increasingly important issue for workers across all age groups, necessitating greater attention to prevention and management strategies in the workplace.

Numerous recent systematic reviews have assessed the effect of interventions aimed at workplace-based prevention and management of musculoskeletal disorders (MSDs) (19–29). Previous reviews have primarily focused on the upper body (19, 21, 23, 26, 27, 29) or specific occupational groups, ie, workers with predominantly physically demanding (25) or sedentary jobs (24, 29). Notably, however, systematic reviews on effectiveness of workplace interventions specifically targeting prevention and/or management of knee pain seem to be lacking.

Consequently, the present systematic review aimed to synthesize the evidence from intervention studies about workplace-based prevention and management of knee pain, thereby providing an evidence-based foundation for the benefit of both employees and employers, occupational health professionals, as well as policy makers.

## Methods

### Study design

This systematic review is reported in accordance with the Preferred Reporting Items for Systematic reviews and Meta-Analyses 2020 (PRISMA 2020) (30) and inspired by the approach of the Institute of Work and Health (IWH) (31). This systematic review was pre-registered in the PROSPERO database under registration number CRD42023350840.

### Eligibility

Studies had to fulfill the following population, intervention, comparison, and outcome (PICO) eligibility criteria to be included in the systematic review. Briefly, we included both randomized (RCT) and non-randomized (non-RCT) studies assessing the effect of any intervention initiated, supported or performed by or at the workplace (I) on knee pain/symptoms/discomfort intensity/severity, -prevalence, or -incidence (O) among currently employed adult workers (P) against any kind of between- or within-subject comparator, ie, receiving no active intervention or usual care/practice (C). Any cause or clinical diagnosis, eg, knee OA, causing the knee-related outcomes were accepted. A broad definition of knee pain was employed, however requiring the explicit reporting of distinct outcome parameters directly reflecting knee pain. Importantly, studies where knee pain outcomes were considered both primary, secondary, as well as a tertiary outcomes were eligible. We excluded studies assessing return-to-work interventions among sick-listed workers, studies without direct between-groups statistical comparison of the outcome of interest, as well as conference abstracts and other references without full data published.

### Literature search

**Figure 1:**
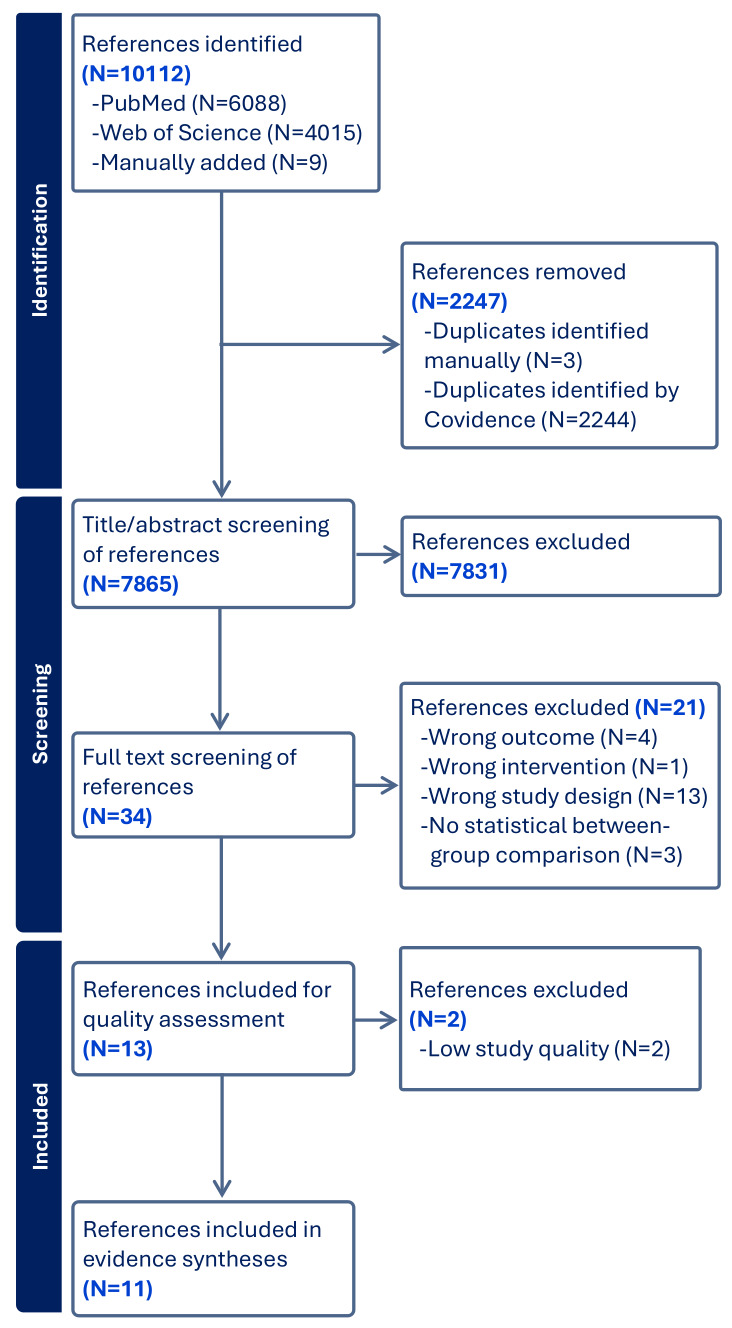
Flowchart.

Using the PICO as guideline, we searched PubMed (including MEDLINE) and Web of Science Core Collection (including 'Science Citation Index Expanded', 'Social Sciences Citation Index' and 'Arts & Humanities Citation Index') systematically for relevant studies published within the last 20 years (2003–2023) and restricted to the following languages: English, Danish, Swedish, and Norwegian. The search strings are reported in the supplementary material (www.sjweh.fi/article/4195) tables S1A-S1B. To capture more relevant studies, we performed additional manual searches using the "snowball" method by pursuing reference lists from key references, including previous reviews. Relevant studies were also identified through the author group's own personal knowledge and communication with experts within the field. A total of 10 112 studies were retrieved, as shown in figure 1.

### Selection process

After removal of duplicates, at least two reviewers independently screened both titles and abstracts of potentially eligible studies using the digital software Covidence (Veritas Health Innovation, Melbourne, Australia). The same procedure was applied for screening of full texts to determine inclusion/exclusion of the study. Disagreements on inclusion/exclusion were resolved through consensus where possible and, where not, a third reviewer acted as arbitrator.

### Data extraction

Following a standardized data extraction form, two reviewers independently extracted data on general characteristics from the included studies, eg, author(s), publication year, sample size etc. These data are reported in supplementary table S2.

### Quality assessment

Inspired by previous IWH- and IWH-inspired systematic reviews (19, 21, 25), two reviewers critically and independently appraised the quality of included records using 17 quality parameters (supplementary table S3). A study was assigned a score of 0 if the quality parameter was not evident, and a score of 1–3 if it was present, with the weighting values for each of the 17 criteria ranging from somewhat important=1 to very important=3. The weighted value reflects the IWH's assessment of the importance of each specific quality parameter. Disagreements were handled through consensus. The quality assessment reflects the internal, external, construct, and statistical validity of each study. Thereby, higher validity typically entails a lower risk of bias. Based on the quality appraisal, each study was given a rank score, which was then divided by the maximal weighted sum score and multiplied by 100 to achieve a percentage score. Studies were subsequently rated as low (<50%), medium (50–85%), or high quality (>85%) depending on their percentage score. The following data extraction and evidence synthesis were only performed on medium and high-quality studies (32).

### Evidence synthesis

IWH's adapted "best evidence synthesis" approach – originally developed by Slavin (32) – was used to synthesize and rate the level of evidence (31) (supplementary table S4). This approach was deemed suitable due to the high heterogeneity in prevention research, particularly within the work environment research field, which tends to make meta-analyses unsuitable. Specifically, the level of evidence is categorized as strong, 'moderate', limited, mixed or insufficient based on the quality and quantity of the included studies, as well as the consistency in the total body of evidence. IWH engaged key stakeholders in defining the key messages for stakeholders accompanying each level of evidence. For instance, a strong level of evidence typically entails a recommendation for practice, whereas a moderate level of evidence leads to a practice consideration (19). Levels of evidence below moderate (ie, limited, mixed, or insufficient) resulted in the following message for practice: "Not enough evidence from the scientific literature to guide current policies/practices". Studies were subsequently grouped into relevant intervention categories and subcategories to allow for tailored evidence syntheses for the benefit of both researchers and stakeholders. Furthermore, studies were stratified into two occupational categories, namely workers/occupational groups with either predominantly physically demanding work tasks or predominantly sedentary work.

## Results

### Study selection

The bibliographical database search covering 2003 to January 2023 identified 10 112 references: 6088 references from PubMed and 4015 from Web of Science. The review group identified and manually added 9 references were identified and manually added through their personal knowledge and using the "snowball" method by pursuing reference lists from key references. After removal of duplicates, 7865 remained for title/abstract screening. During title/abstract screening, 7831 references were excluded, resulting in 34 references for full text retrieval and screening. After full text assessment, 21 studies were excluded for different reasons reported in the PRISMA flowchart (figure 1), leaving 13 studies for quality assessment.

### Quality appraisal

Of the 13 identified studies, 2 were deemed to be low quality (33, 34), 11 medium quality (35–45), and 3 high quality (46–48). Only medium- and high-quality studies were included in the data extraction and evidence synthesis. Ultimately, 11 studies describing a total of 14 interventions (some studies have several intervention arms) for data extraction and evidence synthesis. General characteristics of the included studies are summarized in supplementary table S2.

### Data extraction

Of the eleven studies, seven were RCT (35, 39, 40, 42, 44–48), and two were non-RCT (41, 43). Five studies originated from Denmark (35, 41, 46–48) with the remaining from Iran (39, 43, 45), Canada (42, 44), and Japan (40). Seven studies were among workers with physically demanding jobs (39–41, 45–48) and four included workers with sedentary jobs (35, 42–44). Sample sizes ranged from 24 (42) to 549 (35), and mean age of the sampled workers ranged from 35 (45) to 55 years (42). Three studies included only male workers (39, 45, 46), one study only female workers (47), whereas the remainder included mixed samples. No studies aimed specifically at addressing knee pain, and it was thus not explicitly stated as the primary outcome (supplementary table S2).

### Categorization into different categories

Using a data-driven approach based on the identified studies and applying universally accepted definitions of the intervention category concepts, three broad intervention categories were formed, specifically 'physical exercise/activity' (eight interventions), 'ergonomics' (four interventions) (40, 41, 43, 45) and 'multifaceted' (two interventions) (39, 45). The 'physical exercise/activity' category had three additional subcategories: 'strength exercise', 'aerobic exercise', and 'general physical exercise/activity'). For clarity, 'general physical exercise/activity' was regarded when the exercise component did not fit into the former two categories/exercise modalities, or consisted of a combination of exercise modalities. Likewise, 'ergonomics' was divided into two subcategories: 'ergonomics – few components' and 'ergonomics – multicomponent'. 'Ergonomics – few components' comprised more simple ergonomic workplace-based interventions, eg, provision of tools/equipment, whereas 'ergonomics – multicomponent' workplace-based interventions, ie, comprised both provision of tools/equipment *and* individual and/or organizational level training and supervision/coaching in use of this and/or other ergonomic goals. 'Multifaceted' were defined as workplace-based interventions comprised of multiple different intervention categories, eg 'physical exercise/activity' and 'ergonomics' (supplementary table S2).

### Evidence synthesis

The level of evidence was rated for each intervention category and subcategory across all occupational groups and separately for sedentary work and physically demanding work (table 1), along with an accompanying message to stakeholders. As none of the included studies aimed specifically at addressing knee pain, making direct recommendations to stakeholders should be done with great caution. The following messages to stakeholders should be read with that in mind, as they were based on the currently limited available studies which primarily assess knee pain as a secondary/tertiary outcome.

**Table 1:** Evidence synthesis. [M=medium-quality study; H=high-quality study]

Intervention (sub)category		Studies	Interventions	Consistency	Level of evidence	Message for stakeholders
Physical exercise/activity		7	8	1 Effect (M=1) 7 No effect (H=3, M=4)	Strong (of no effect)	Workplace-based physical exercise/activity not specifically aimed at addressing knee pain have not successfully prevented/managed knee pain
Strength training		4	4	1 Effect (M=1) 3 No effect (H=1, M=2)	Moderate (of no effect)	Workplace-based strength training not specifically aimed at addressing knee pain have not successfully prevented/managed knee pain
Aerobic exercise		1	1	0 Effect 1 No effect (H=1)	Limited (of no effect)	Not enough evidence from the scientific literature to guide current policies/practices
General physical exercise/activity		3	3	0 Effect 3 No effect (H=1, 2=M)	Moderate (of no effect)	Workplace-based general physical exercise/activity not specifically aimed at addressing knee pain have not successfully prevented/managed knee pain
Ergonomics		4	4	1 Effect (M=1) 3 No effect (M=3)	Limited (of no effect)	Not enough evidence from the scientific literature to guide current policies/practices
Ergonomics - few components		2	2	0 Effect 2 No effect (M=2)	Limited (of no effect)	Not enough evidence from the scientific literature to guide current policies/practices
Ergonomics - multicomponent		2	2	1 Effect (M=1) 1 No effect (M=1)	Mixed	Not enough evidence from the scientific literature to guide current policies/practices
Multifaceted		2	2	2 Effect (M=2, 0 No effect	Limited (of effect)	Not enough evidence from the scientific literature to guide current policies/practices
**Sedentary workers**						
	Physical exercise/activity	3	4	1 Effect (M=10 3 No effect (M=3)	Limited (of no effect)	Not enough evidence from the scientific literature to guide current policies/practices
	Strength training	3	3	1 Effect (M=1) 2 No effect (M=2)	Limited (of no effect)	Not enough evidence from the scientific literature to guide current policies/practices
	General physical exercise/activity	1	1	0 Effect 1 No effect (M=1)	Insufficient	Not enough evidence from the scientific literature to guide current policies/practices
	Ergonomics	1	1	1 Effect (M=1) 0 No effect	Insufficient	Not enough evidence from the scientific literature to guide current policies/practices
	Ergonomics - few components	1	1	1 Effect (M=1) 0 No effect	Insufficient	Not enough evidence from the scientific literature to guide current policies/practices
**Physically ** **demanding work**						
	Physical exercise/activity	4	4	0 Effect 4 No effect (H=3, M=1)	Strong (of no effect)	Workplace-based physical exercise/activity not specifically aimed at addressing knee pain have not successfully prevented/managed knee pain
	Strength training	1	1	0 Effect 1 No effect (H=1)	Limited (of no effect)	Not enough evidence from the scientific literature to guide current policies/practices
	General physical exercise/activity	2	2	0 Effect 2 No effect (H=1, M=1)	Limited (of no effect)	Not enough evidence from the scientific literature to guide current policies/practices
	Aerobic exercise	1	1	0 Effect 1 No effect (H=1)	Limited (of no effect)	Not enough evidence from the scientific literature to guide current policies/practices
	Ergonomics	3	3	0 Effect 3 No effects (M=3)	Limited (of no effect)	Not enough evidence from the scientific literature to guide current policies/practices
	Ergonomics - few components	2	2	0 Effect 2 No effect (M=2)	Limited (of no effect)	Not enough evidence from the scientific literature to guide current policies/practices
	Ergonomics - multicomponent	1	1	0 Effect 1 No effect (M=1)	Limited (of no effect)	Not enough evidence from the scientific literature to guide current policies/practices
	Multifaceted	2	2	2 Effect (M=2) 0 No effect	Limited (of effect)	Not enough evidence from the scientific literature to guide current policies/practices

### All occupations

*Physical exercise/activity.* Seven studies reported on eight interventions concerning physical exercise/activity (35, 42, 44–48) and were further subcategorized into 'strength training', 'aerobic exercise' and 'general physical exercise/activity' (supplementary table S2 and table 1). In the main category 'physical exercise/activity', eight interventions from three high-quality studies (46–48) and four medium-quality studies (35, 42, 44, 45) attained a strong level of evidence for no effect on knee pain. This led to the following message for stakeholders: "Workplace-based physical exercise/activity programs not specifically aimed at addressing knee pain have not successfully prevented/managed knee pain".

Four studies and accompanying four interventions on the subcategory 'strength training' were identified (35, 42, 44, 47), including one intervention from a high-quality study (47) and three interventions from medium-quality studies (35, 42, 44). These studies formed a moderate level of evidence for no effect on knee pain, leading to the following message for stakeholders: "Workplace-based strength training not specifically aimed at addressing knee pain have not successfully prevented/managed knee pain".

One high-quality study reporting on one 'aerobic exercise' intervention was identified (48) and provided a limited level of evidence of no effect on knee pain, leading to the following message for stakeholders: "Not enough evidence from the scientific literature to guide current policies/practices".

Finally, three interventions from one high-quality study (46) and two medium-quality studies (35, 45) reporting on 'general physical exercise/activity' achieved a moderate level of evidence for no effect on knee pain, resulting in the following message for stakeholders: "Workplace-based general physical exercise/activity not specifically aimed at addressing knee pain have not successfully prevented/managed knee pain".

*Ergonomics.* A total of four moderate-quality studies reporting on four interventions assessed the effect of ergonomic interventions on knee pain (40, 41, 43, 45) and yielded a limited level of evidence for no effect on knee pain for the 'ergonomics' category. Two studies subcategorized into 'ergonomics – few components' (40, 45) constituted a limited level of evidence for no effect on knee pain, whereas two other studies subcategorized into 'ergonomics – multicomponent' (41, 43) provided a mixed level of evidence in terms of the effect on knee pain. The following message for stakeholders pertained both the overarching 'ergonomics' category and the two subcategories: "Not enough evidence from the scientific literature to guide current policies/practices".

*Multifaceted.* Two medium-quality studies reporting on two multifaceted interventions provided a limited level of evidence for an effect on knee pain (39, 45), resulting in the following message for stakeholders: "Not enough evidence from the scientific literature to guide current policies/practices".

### Occupation-specific categorization

*Sedentary work.* A total of four medium-quality studies reporting on five interventions were identified across intervention categories among workers with predominantly sedentary jobs (35, 42–44). Within this occupational group, the level of evidence varied from insufficient to limited across all categories and subcategories, resulting in the following message for stakeholders: "Not enough evidence from the scientific literature to guide current policies/practices".

*Physically demanding work.* Nine studies reporting on nine interventions were identified across intervention categories among workers with predominantly physically demanding work (39–41, 45–48). A strong level of evidence was found for no effect of the workplace-based 'physical exercise/activity' category, leading to the following message for stakeholders: "Workplace-based physical exercise/activity programs not specifically aimed at addressing knee pain have not successfully prevented/managed knee pain".

Two medium-quality studies reporting two multifaceted workplace-based interventions reported an effect on knee pain. However, for this and the other remaining categories and subcategories, the level of evidence was deemed limited, resulting in the following message for stakeholders: "Not enough evidence from the scientific literature to guide current policies/practices".

## Discussion

In the present systematic review including 11 medium- and high-quality studies, neither workplace-based physical exercise/activity programs nor ergonomic initiatives seemed effective for preventing and/or managing knee pain. Two medium-quality studies suggested that multifaceted interventions could be effective for knee pain. Still, based on the current limited level of evidence where no studies specifically aimed at knee pain, no workplace-based interventions can be recommended for stakeholders for effective prevention and management of knee pain, a condition with massive multi-level consequences.

To inform development and implementation of preventive efforts at the workplaces and proper policy development, more high-quality research on workplace-based interventions with the primary aim of prevention and management of knee pain is warranted.

### Physical exercise/activity

Among the included studies predominantly having knee pain as a secondary/tertiary outcome, the present systematic review found strong evidence for no effect of workplace-based physical exercise/activity on knee pain across occupational categories and specifically among workers with physically demanding jobs. This finding is surprising/unexpected as other recent systematic reviews have found workplace-based physical exercise/activity to be effective in reducing non-specific musculoskeletal pain and pain in the neck and lower back (19–23, 25). Workplace-based physical exercise/activity has also been associated with a reduced risk of long-term sickness absence (46). Furthermore, strong evidence exists for an effect of leisure-time exercise on knee pain/OA (49), and exercise/physical activity remains a core component of leading authorities' recommendations for the non-pharmacological/non-surgical management of knee OA (17, 50). Importantly, though, none of the physical exercise/activity interventions yielded negative effects on knee pain, and several of the included interventions demonstrated positive effects on other outcomes (some reported in other publications), ie, reduced low-back pain (35, 40, 47), improved objective physical performance (44, 46–48), and positive effects on work ability (47, 48) and self-reported lower extremity function (44).

Several plausible explanations for the unexpected findings regarding the lack of effect of workplace-based physical exercise/activity on knee pain are worth mentioning. First, knee pain was not an explicitly stated primary outcome of any of the included studies, and hence the studies were not specifically designed to investigate this outcome. For example, several of the included studies actually achieved significant improvements in their stated primary outcome, ie, low-back pain (40, 47) and lower extremity function (44). Second, although no one-size-fits-all 'optimal' exercise prescription for, eg, knee OA seems to exist (51), a plausible explanation for the present lack of measurable effect on knee pain from workplace-based physical exercise/activity could be suboptimal exercise protocols in terms of, eg, type, duration, frequency, dosage, and loading intensity (FITT principles). However, reporting transparency regarding adherence and implementation was generally suboptimal in the included studies. Still, all included studies, with the exception of that of Andersen and colleagues (35), which solely focusing on neck and shoulder exercises, targeted the lower extremities. Most reported somewhat satisfactory adherence, for which reason the exercise interventions overall seem suitable for improving knee joint health. Importantly, it is increasingly recognized that the exercise-induced improvements in knee pain experienced by many knee OA patients are largely driven by non-specific factors, ie, contextual factors, placebo responses, the natural course of the disease, and the regression to the mean phenomenon (49). In addition, and adhering to the multifactorial etiology of knee pain, changes in other work factors previously shown to influence pain, eg, supervisor and collegial support (52), may further have confounded the (lack of) effect of workplace-based physical exercise/activity on knee pain.

Since baseline pain intensity has been shown to be an important moderator of the therapeutic effect of exercise (49), another possible explanation for the lack of effect could relate to the baseline knee pain intensity of the participating workers. Most included studies did not specify knee pain/symptoms/intensity as inclusion criteria and thus included heterogenic samples of workers with varying degrees of knee pain, ie, both asymptomatic and symptomatic. In this case, subgroup/sensitivity analysis only including symptomatic participants with higher baseline knee pain could have provided valuable information (35, 53).

### 'Ergonomics' and 'multifaceted' interventions

For the intervention categories 'ergonomics' and 'multifaceted', the overall level of evidence was deemed too uncertain, specifically limited, mixed, or insufficient, to guide current policies/practices on workplace-based prevention and management of knee pain. The scientific support for ergonomic interventions from previous recent systematic reviews and randomized controlled trials is mixed, with some suggestions of a positive effect on upper limb pain (23) and others no effect on pain in different body regions (20, 25, 54, 55). Specifically pertaining to knee pain, a recent paper reported a health-impact assessment of a newly-developed assistive device (a manually movable screed levelling machine), showing promising potential in reducing knee joint loading and thereby the risk of knee OA among floor layers compared to traditional working techniques (56). This finding by Kuijer et al is in line with previous work by Jensen & Friche, showing short- and long-term reduced risk of knee complaints from an altered working technique resulting from a comprehensive participatory ergonomics implementation strategy (57–59). Additionally, two ergonomics interventions did not find positive effects on knee pain (60, 61). Collectively, the effectiveness of ergonomics interventions on prevention and management of knee pain seems questionable.

Granted the multifactorial nature of knee pain, workplace-based prevention and management of knee pain calls for multifaceted workplace interventions adhering to a biopsychosocial approach encompassing both mainly ergonomic/physical factors, eg, workstation adjustments, use of assistive devices, or physical exercise, *and* psychosocial components, ie, stress management and/or supervisor or collegial support (62). Only two studies reporting on two multifaceted workplace-based interventions were included in the present review, and both study interventions addressed predominantly ergonomic/physical components, namely the combination of ergonomics and physical exercise. Both studies reported a positive effect on knee pain and were performed among workers with physically demanding work. Acknowledging the limited number of studies applying multifaceted interventions in the current literature, this could suggest a potential in multifaceted approaches for workplace-based prevention and management of knee pain that are worth exploring in future trials. Nonetheless, Sundstrup and colleagues recently reported no effect on pain (not knee pain alone) as result of multifaceted workplace interventions among workers with physically demanding jobs (25). Likewise, Szeto et al (63) reported no effect on knee/thigh function of a multifaceted 8-week workplace intervention consisting of both ergonomic training and physical exercise compared to a control group receiving no intervention. In summary, more research is needed to assess the effectiveness of multifaceted workplace-based interventions on knee pain.

### Quality appraisal

Aside from the mere paucity of studies of sufficient quality to enter the evidence synthesis, the quality appraisal process revealed several limitations of the included medium- and high-quality studies that are worth addressing in future studies. The most prominent included short follow-ups (<3 months), inadequate detail and transparency in reporting of group allocation procedures, recruitment rate, intervention processes, and participation rate, as well as inadequate performance and/or reporting of statistical analyses, eg, lack of dropout analyses and analyses based on the intention-to-treat principles etc. For instance, many studies did not report recruitment rate or performed formal dropout analyses, which ie, challenges assessment of the risk of selection bias. In addition, very few studies documented potential effects on exposure parameters, which could have given indications of potentially mediating factors.

Notably, none of the included studies explicitly stated knee pain as the primary outcome. Knee pain outcomes were typically reported as secondary and tertiary outcomes. As such, studies were not specifically methodologically designed for the assessment of changes in knee pain. This fact can also partly explain the overall low quality of the statistical analyses with respect to knee pain outcomes, ie, the lack of adjustment for pre-intervention differences in knee pain and intention-to-treat analyses.

Another limitation pertains to the dynamic and highly variable trajectory of knee pain within and between days and individuals and, thereby, the included studies' follow-up lengths. It is possible that meaningful changes in knee pain were not fully captured, especially not in studies with few and/or shorter follow-ups. In addition, workplace interventions are complex, and it can take considerable time and resources to successfully implement new workplace policies or practices. The included studies were also characterized by limited reporting of implementation-specific/process evaluation outcomes, ie, fidelity, adherence and otherwise participation rate. It has been noted by others previously that the occupational health and safety research field has been lacking focus on implementation science, which can have hindered uptake and hence effectiveness of evidence-based workplace interventions on (knee) pain (21, 25, 64).

### Strength and limitations of the current systematic review

This IWH-inspired systematic review sought to provide actionable recommendations for workplace practice about a largely neglected area of work environment research on a health condition with major global impact, specifically knee pain. Still, several limitations of this review are worth noting.

First, the present systematic review applied and systematically searched for a broad definition of knee pain and excluded studies not specifically addressing knee pain but other relevant knee-related concepts, eg, overall knee/thigh function (63). Searching for knee pain alone could hence have left out potentially relevant information about eg, knee-related (work) disability and sickness absence. However, direct study exclusion on this criteria was very seldom (63). In addition, as knee pain was not an explicitly stated primary outcome of any of the included studies, it is possible that relevant studies were excluded during the screening process if the reporting of knee pain outcomes were not properly expressed. This also explains why a considerable number of the included studies were manually added by the author group based on insights into the topic and not identified through the systematic literature search.

Publication bias is a risk as we did not search for grey literature that could have provided valuable information not available in published peer-reviewed papers. The inclusion of (two) non-RCT comes with both strengths and limitations. The RCT remains the golden standard for establishing cause-and-effect, and non-RCT thus entail an increased risk of bias. To address this, we adopted the IWH approach for quality assessment and subsequent evidence synthesis, which is specifically designed for assessing study designs other than RCT. As highlighted previously (21, 25), solely including RCT that are restrictive, highly resource demanding and hence not always feasible in workplace settings would thus restrict our understanding of effective workplace interventions by neglecting valuable information from comparably more feasible non-RCT. We did not perform a meta-analysis due to heterogeneity of the knee pain outcomes and performed statistics. Still, we think the practical value of formulating key messages for stakeholders is an important methodological strength of this review, although the evidence largely showed too limited to provide actionable recommendations. Although the majority of included studies were from Scandinavia, specifically Denmark, we also included several studies from other parts of the world, namely Iran, Canada and Canada. Most of the included studies focused on health-related professions, eg, healthcare workers and dentists. It is plausible that the findings' generalizability would be higher if more studies from a more diverse representation of countries and occupational groups had been identified and included. Another strength of this study is that at least two review team members independently performed every step of the review process.

### Implications for practice, policy, and future research

This systematic review demonstrates a paucity of and overall shortcomings in the current evidence-base pertaining workplace-based prevention and management of knee pain. Therefore, it is currently not possible to provide trustworthy and actionable recommendations for effective preventive practice or policy that are based on strong scientific evidence. Hence, more high-quality research is clearly needed on work-related risk factors and protective factors for knee pain and specific preventive interventions aimed at tackling this.

For instance, multiple work-related risk factors for knee pain have been identified (12), but to this day it remains largely unknown how, eg, ergonomic and psychosocial workplace factors interact and how this knowledge can and should be transferred into preventive practice. The low number of included studies in this systematic review and the fact that knee pain was a secondary/tertiary outcome in the majority of the studies emphasize the eminent need for more high-quality interventional research assessing concrete preventive efforts aimed specifically at knee pain and its related consequences, eg, in form of randomized controlled trials with longer follow-ups assessing integrated biopsychosocial and organizationally anchored workplace interventions among ie, high-risk working populations such as construction workers (12). Importantly, knee pain is influenced by a complex interplay of modifiable and non-modifiable factors, including both occupational and non-occupational factors (11–15). While workplace-based interventions can target reduction of occupational risk factors, eg, reducing high occupational physical workloads, and promote protective factors, eg, physical exercise, workplace-based preventive efforts should be supplemented with strategies addressing personal and leisure-time factors to optimize overall prevention.

### Concluding remarks

In the present systematic review, neither previous workplace-based physical exercise/activity nor ergonomic interventions seemed effective for prevention and/or management of knee pain. A limited level of evidence suggested that multifaceted interventions could be effective for knee pain.

Still, the current evidence-base pertaining for workplace-based prevention and management of knee pain remains too limited to guide effective preventive workplace practice or policy. There is a pressing need to develop effective workplace interventions to prevent and manage knee pain – a significant global health issue projected to rise in prevalence in the coming years.

## Supplementary material

Supplementary material

## Data Availability

Data in this review will be available from the corresponding author upon reasonable request.
