# Supplementary material for: Workplace-based prevention and management of knee pain: a systematic review
Source: Scand J Work Environ Health. 2024 Dec 30;51(1):3–14. doi: 10.5271/sjweh.4195 (PMC11696606; doi:10.5271/sjweh.4195)
Supplement: Supplementary material [file SJWEH-51-3-S001.pdf]

Workplace-based prevention and management of knee pain: a systematic review[1]

by Sebastian Venge Skovlund, MSc,[2] Mark Skovbye Eg Østergaard, MSc, Karina GV Seeberg, MSc, Charlotte Suetta, PhD, Per Aagaard, PhD, Lars Louis Andersen, PhD, Emil Sundstrup, PhD [1]

1. Supplementary Material
2. Correspondence to: Sebastian Venge Skovlund, National Research Centre for the Working Environment, Lersø Parkallé 105, 2100 Copenhagen, Denmark. [E-mail: svs@nfa.dk]

Supplementary Table S1A. PubMed search string.

(Occupations [Mesh] OR Work Performance [Mesh] OR Work [Mesh] OR Employment [Mesh] OR Occupational Health [Mesh] OR Occupational diseases [Mesh] OR Workplace [Mesh] OR Workforce [Mesh] OR Employ* [Title/Abstract] OR Healthcare industr* [Title/Abstract] OR Health care industr* [Title/Abstract] OR Labor* [Title/Abstract] OR Labour* [Title/Abstract] OR Blue collar [Title/Abstract] OR Blue-collar [Title/Abstract] OR White collar [Title/Abstract] OR White-collar [Title/Abstract] OR Wage earner* [Title/Abstract] OR Occupation* [Title/Abstract] OR Job [Title/Abstract] OR Jobs [Title/Abstract] OR Work* [Title/Abstract] OR Vocation* [Title/Abstract] OR Claimant* [Title/Abstract])<br><br>AND (Patellofemoral Pain Syndrome [Mesh] OR Knee Injuries [Mesh] OR Osteoarthritis, Knee [Mesh] OR Tibial Meniscus Injuries [Mesh] OR (Knee [Title/Abstract] AND (pain [Title/Abstract] OR discomfort[Title/Abstract] OR ache[Title/Abstract] OR sore*[Title/Abstract] OR injur*[Title/Abstract] OR symptom* [Title/Abstract] OR disorder* [Title/Abstract] OR problem* [Title/Abstract] OR osteoarthritis [Title/Abstract] OR arthritis [Title/Abstract] OR arthrosis [Title/Abstract])) OR Patellofemoral osteoarthritis [Title/Abstract] OR Patello-femoral osteoarthritis [Title/Abstract] OR Tibiofemoral osteoarthritis [Title/Abstract] OR Tibio-femoral osteoarthritis [Title/Abstract])<br><br>AND (Accident prevention [Title/Abstract] OR advice [Title/Abstract] OR back school [Title/Abstract] OR engineering design/redesign [Title/Abstract] OR postur* [Title/Abstract] OR surveillance [Title/Abstract] OR screening[Title/Abstract] OR adjust* [Title/Abstract] OR assist* [Title/Abstract] OR cloth* [Title/Abstract] OR device [Title/Abstract] OR knee brace [Title/Abstract] OR insole [Title/Abstract] OR lumbosacral support [Title/Abstract] OR lumbar support [Title/Abstract] OR exoskeleton [Title/Abstract] OR physiotherap* [Title/Abstract] OR occupational therap* [Title/Abstract] OR onsite treatment [Title/Abstract] OR Health promotion[Title/Abstract] OR Healthcare provider training[Title/Abstract] OR Human resource [Title/Abstract] OR Intervention*[Title/Abstract] OR injury prevention [Title/Abstract] OR injury control [Title/Abstract] OR injury assessment

[Title/Abstract] OR accommodation[Title/Abstract] OR Legislation enforcement[Title/Abstract] OR Light duty [Title/Abstract] OR Light duties [Title/Abstract] OR Modified duty [Title/Abstract] OR Modified duties [Title/Abstract] OR Modified job[Title/Abstract] OR Modified work[Title/Abstract] OR Modified tasks[Title/Abstract] OR Multidisciplinary [Title/Abstract] OR Guidelines [Title/Abstract] OR management [Title/Abstract] OR medicine [Title/Abstract] OR rehabilitation [Title/Abstract] OR Organi* [Title/Abstract] OR OSH [Title/Abstract] OR OHS [Title/Abstract] OR Pain reduction[Title/Abstract] OR Participatory [Title/Abstract] OR physical therap*[Title/Abstract] OR prevention and control[Title/Abstract] OR prevention strateg*[Title/Abstract] OR Psychotherap*[Title/Abstract] OR Rest break* [Title/Abstract] OR Secondary prevention [Title/Abstract] OR Self-management [Title/Abstract] OR Service coordination [Title/Abstract] OR Stress management [Title/Abstract] OR Structured rehabilitation program [Title/Abstract] OR Suitable duty [Title/Abstract] OR Suitable duties [Title/Abstract] OR Supportive colleagues[Title/Abstract] OR Supportive manager[Title/Abstract] OR collegial support [Title/Abstract] OR managerial support [Title/Abstract] OR safety climate [Title/Abstract] OR safety culture [Title/Abstract] OR safety incentive [Title/Abstract] OR Technical change*[Title/Abstract] OR Training[Title/Abstract] OR Education*[Title/Abstract] OR Protect* [Title/Abstract] OR Assessment [Title/Abstract] OR Rehabilitation [Title/Abstract] OR Conditioning [Title/Abstract] OR disability management [Title/Abstract] OR hardening[Title/Abstract] OR program*[Title/Abstract] OR trial*[Title/Abstract] OR modifi*[Title/Abstract] OR learning [Title/Abstract] OR visit* [Title/Abstract] OR Absence management [Title/Abstract] OR Adequate supervision [Title/Abstract] OR Alternative [Title/Abstract] OR Attendance management [Title/Abstract] OR Behav* [Title/Abstract] OR Biofeedback [Title/Abstract] OR CBT [Title/Abstract] OR Combined modality therap*[Title/Abstract] OR Compensation management[Title/Abstract] OR Complementary Therap*[Title/Abstract] OR Coordinated [Title/Abstract] OR Counselling [Title/Abstract] OR Disclosure management [Title/Abstract] OR Contact [Title/Abstract] OR Environment* [Title/Abstract] OR Ergonomic* [Title/Abstract] OR Exercise*[Title/Abstract] OR Flexible [Title/Abstract] OR Functional abilit* [Title/Abstract] OR Functional capacit* [Title/Abstract] OR Good practice[Title/Abstract] OR graded activit*[Title/Abstract] OR Graded [Title/Abstract] OR Health care provider [Title/Abstract] OR motivat*[Title/Abstract] OR Psychosocial* [Title/Abstract] OR stress prevention [Title/Abstract] OR resident handling [Title/Abstract])
AND ((2003:3000/12/12[pdat])
AND (danish[Filter] OR english[Filter] OR norwegian[Filter] OR swedish[Filter]))

Supplementary Table S1B. Web of Science search string.

(TS=(occupation* or “work-related” or “working environment*” or “work environment*” or workplace* or “work place*” or worksite* or “work site*” or workload or “work load” or “at work” or work* or “work condition*” or job* or employ* or industr* or Labo$r or labo$rer or “blue collar” or “blue-collar” or “white collar” or “white-collar” or “wage earner$” OR vocation* or “work force” or workforce* or claimant*)) AND<br><br>((TS=(knee$ near (pain or discomfort* or complaint* or injur* or disorder* or symptom* or degenerative or coxarthrosis or arthriti* or arthrosis or gonarthrosis or osteoarthriti* OR osteo-arthriti* OR osteoarthro*))) OR<br>(TS=(*femoral near (pain or discomfort* or complaint* or injur* or disorder* or symptom* or degenerative or coxarthrosis or arthriti* or arthrosis or osteoarthriti* OR osteo-arthriti* OR osteoarthro*)))) AND<br><br>(TS=(“accident prevention” OR advice* or “back school” OR “engineering design/redesign” OR “employee assistance program” OR postur* OR surveillance OR screening OR adjust* OR assist* OR cloth* or device OR “knee brace” OR insole OR “lumbosacral support” OR “lumbar support” OR exoskeleton OR physiotherap* OR “occupational therap*” OR “onsite treatment” OR “Health promotion” OR “Healthcare provider training” OR “Human resource” OR intervention* OR “injury prevention” OR “injury control” OR “injury assessment” OR accommodat* OR “Legislation enforcement” OR “Light duty” OR “Light duties” OR “Modified duty” OR<br>“Modified duties” OR “Modified job” OR “Modified work” OR<br>“modified tasks” OR Multidisciplinary OR Guideline* OR management OR<br>medicine OR rehabilitat* OR Organi* OR<br>OSH OR OHS OR “Pain reduction” OR Participat* OR “physical therap*” OR<br>“prevention and control” OR “prevention strateg*” OR Psychotherap* OR<br>“Rest break*” OR “Secondary prevention” OR “Self-management” OR<br>“service coordination” OR “Stress management” OR “Structured rehabilitation program” OR<br>“Suitable duty” OR “suitable duties” OR<br>“Supportive colleagues” OR “Supportive manager” OR “collegial support” OR “managerial support” OR “safety climate” OR<br>“safety culture” OR “safety incentive program*” OR “Technical change*” OR<br>Training OR Education* OR Protect* OR Assessment OR Rehabilitation OR<br>Conditioning OR “disability management” OR hardening OR program* OR<br>trial* OR modifi* OR learning OR visit* OR “Absence management” OR “Adequate supervision” OR Alternative OR “Attendance management” OR<br>Behav* OR Biofeedback OR CBT OR “Combined modality therap*” OR<br>“Compensation management” OR “Complementary Therap*” OR<br>Coordinated OR counselling OR “Disclosure management” OR<br>Assist* OR Contact OR Environment* OR Ergonomic* OR<br>Exercise* OR Flexible OR “Functional abilit*” OR “Functional capacit*” OR<br>“Good practice” OR Graded OR “Health care provider” OR motivat* OR Psychosocial* OR “stress prevention” OR “resident handling ”)) and 2023 or 2003 or 2004 or 2005 or 2006 or 2007 or 2008 or 2009 or 2010 or 2011 or 2022 or 2021 or 2020 or 2019 or 2018 or 2017 or 2016 or 2015 or 2014 or 2013 or 2012 (Publication Years) and English (Languages)

Supplementary Table S2. Study characteristics.

Author (year) country	Study design	N	Population	Age (SD) (years)	Sex/gender	Follow-up	Groups	Study quality	Primary outcome	Knee pain outcome at baseline	Positive effect on knee pain
**Physically demanding work**											
**Ergonomics**											
Ergonomics - few components											
Hagiwara (2017) Japan	RCT	119	Healthcare workers	I = 44.7 (10). C = 44.7 (9.6)	All: M = 3, F = 104. I: M = 2. F = 52. C: M = 1. F = 53	3 months	I = spinal underwear for lumbosacral support<br>C = no intervention	medium	Lower back pain (VAS)	Knee pain prevalence<br>I = 12 (22.2%)<br>C = 16 (30.2%)	No
Mousavi (2019) Iran	RCT	100	Production factory workers	Ia = 35 (3.6). C = 36 (5.6)	M	8 weeks	Ia = insoles<br>C = no intervention	medium	Lower back and lower limb discomfort	Knee discomfort/ pain (VAS 0-100 mm):<br>Ia = 25.2<br>C = 18.4	No
Ergonomics – multicomponent											
Risør (2017) Denmark	Non-RCT	494	Healthcare workers	Mean age not reported - only age group-	All: M = 32. F = 460. I: M = 22. F = 269. C: M = 10. F = 191	12 months	I = funds to purchase, guidelines and training in use of (new) patient-handling equipment<br>C = usual practice	medium	Appropiate use of patient-handling equipment	Prevalence of knee pain/problems last 12 months and 7 days	No

				specific numbers /frequencies						12 months: I = 27.8% C = 26.1% 7 days: I = 45.7% C = 36.5%	
**Physical exercise/activity**											
Aerobic exercise											
Korshøj (2018) Denmark	cRCT	116	Cleaning assistants	All: 45.3 (8.6)	All: M = 28. F = 88. I: M = 14. F = 43. C: M = 14. F = 45	4 and 12 months	I = aerobic exercise C = no intervention besides lectures about healthy living	high	Cardiorespiratory fitness	Highest Knee pain intensity last month, NMQ (NRS 0-10) at 4 and 12 months. 4 months: I = 4.08 C = 4.15 12 months: I = 3.69 C = 3.83	No
Strength training											
Jakobsen (2018) Denmark	cRCT	200	Healthcare workers	I = 40 (12). C = 44 (10)	F	10 weeks	I = workplace full-body strength training C = home-based full-body strength training	high	Lower back, neck, and shoulder pain	Knee pain intensity last week (modified VAS 0-10): I = 0.9	No

								(intensity)	C = 1.7	
General physical exercise/activity										
Gram (2012) Denmark	RCT	67	Construction workers	All = 43.7 (10.5): I = 44 (11.1). C = 43 (10.0)	M	12 weeks	I = Aerobic and strength training<br>C = 1-hour lecture on general health promotion	high	VO2 max and isometric muscle strength	Knee pain intensity last 7 days, NMQ (NRS 0–10):<br>I = 1.9<br>C = 1.6	No
Mousavi (2019) Iran	RCT	100	Production factory workers	Ib = 36 (4.8). C = 36 (5.6)	M	8 weeks	Ib = exercise and stretching<br>C = no intervention	mediu m	Lower back and lower limb discomfo rt	Knee discomfort (VAS 0-100 mm):<br>:<br>Ib = 21.8<br>C = 18.4	No
**Multifaceted**										
Dehghan (2016) Iran	RCT	102	Dentists	I = 39.8 (4.6). C = 40.0 (4.1)	M	3 and 6 months	I = Ergonomics and stretches<br>C = no intervention	mediu m	Prevalen ce of musculos keletal disorders in general	Prevalence of knee pain, NMQ:<br>I = 15%<br>C = 16%	Yes
Mousavi (2019) Iran	RCT	100	Production factory workers	Ic = 36 (4.7). C = 36 (5.6)	M	8 weeks	Ic = insoles + exercise and stretching<br>C = no intervention	mediu m	Lower back and lower	Knee discomfort	Yes

									limb discomfo rt	(VAS 0-100 mm):<br><br>Ic = 18.4<br>C = 18.4	
**Sedentary work**											
**Ergonomics**											
Ergonomics – multicomponent											
Khalili (2018)<br>Iran	Non-RCT	176	University employees, computer users	I = 39.5 (8.8). C = 35.1 (7.4)	M = 67. F = 109	3 months	I = ergonomic training<br>C = no intervention	mediu m	Practicin g correct body postures	Prevalence of knee pain, NMQ:<br>I = 24%<br>C = 28%	Yes
**Physical exercise/activity**											
Strength training											

Andersen (2010) Denmark	RCT	549	Office workers	Ia: M = 47.3 (9.3). F = 45.5 (10.4). C: M = 46.3 (9.0). F = 43.9 (9.7)	All: M = 195. F = 354. Ia: M = 54. F = 126. C: M = 74. F = 108	1 year	Ia = Strength training of neck and shoulder muscles<br>C = General health counselling and encouragement of organizational health initiatives	mediu m	Neck/sho ulder pain and maximal muscle strength	Knee pain intensity during the last three months, NMQ (scale from 0 to 9):<br>Ia = NA<br>C = NA	No
Chopp-Hurley (2017) Canada	RCT	24	University workers from a variety of occupations (predominan tly sedentary)	I = 52.8 (6.4). C = 54.9 (6.7)	All: M = 5. F = 19. I: M = 2. F = 10. C: M = 3. F = 9	12 weeks	I = leg-strengthening exercise<br>C = maintain current physical activity level	mediu m	Work ability and resilience	KOOS pain subscale (0-100):<br>I = NA<br>C = NA	Yes
Mulla (2018) Canada	RCT	43	Office workers	I = 44.1 (10.5). C = 43.3 (10.4)	All: M = 16. F = 27. I: M = 9. F = 12. C: M = 7. F = 15	12 weeks	I = leg-strengthening exercise<br>C = maintain current physical activity level	mediu m	Lower Extremity Function al Scale	KOOS pain subscale (0-100):<br>I = 92.2<br>C = 94.7	No
General physical exercise/activity											

Andersen (2010) Denmark	RCT	549	Office workers	Ib: M = 43.1 (9.5). F = 44.4 (8.0). C: M = 46.3 (9.0). F = 43.9 (9.7)	All: M = 195. F = 354. Ib: M = 67. F = 120. C: M = 74. F = 108	1 year	Ib = Increase daily activity level<br>C = General health counselling and encouragement of organizational health initiatives	mediu m	Neck/sho ulder pain and maximal muscle strength	Knee pain intensity during the last three months, NMQ (scale from 0 to 9):<br>Ib = NA<br>C = NA	No

N=number; M=Males; F=Females; I=Intervention; Ia-c=Intervention a, b, and c; C=Control; non-RCT=non-randomized controlled trial; cRCT=cluster-randomized controlled trial; RCT=randomized controlled trial; NRS=Numeric rating scale; VAS=Visual analog scale; NMQ=Nordic Musculoskeletal Questionnaire; KOOS=Knee injury and Osteoarthritis Outcome Score

Supplementary Table S3. Quality assessment parameters.

**Question**	**Weight/Points per question**
1. Is the research question clearly stated?	2
2. Were comparison group(s) used?	3
3. Was an intervention allocation described adequately?	3
4. Was the intervention allocation randomized?	3
5. Was recruitment (or participation) rate reported?	2
6. Were pre-intervention characteristics described?	2
7. Was loss to follow-up (attrition)<35%?	2
8. Did the author examine for important differences between the remaining and drop-out participants after the intervention?	2
9. Was the intervention process adequately described to allow for replication?	3
10. Were the effects of the intervention on some exposure parameters documented?	1
11. Was the participation in the intervention documented?	2
12. Were knee pain, symptoms, discomfort and/or disorders described at baseline and at follow-up?	3
13. Was the length of follow-up three months or greater?	2
14. Was there adjustment for pre-intervention diferences (minimum threshold of three important covariates include age, gender and primary outcome at baseline)?	3
15. Were the statistical analyses optimized for the best results?	3
16. Were all participants' outcomes analyzed by the groups to which they were originally allocated (intention-to-treat analysis)?	2
17. Was there a direct between-group comparison?	3

Supplementary Table S4. Best evidence synthesis guidelines.

Level of evidence	Minimum quality	Minimum quantity	Consistency	Terminology for messages
**Strong**	High (> 85%)	Three	Three high-quality studies agree<br><br>If more than three studies, 3/4th of the medium and high-quality studies agree	Recommendations
**Moderate**	Medium (50–85%)	Two high-quality OR Two medium-quality and one high-quality	Two high-quality studies agree OR<br><br>Two medium-quality studies and one high-quality study agree. If more than three studies, more than 2/3rd of the medium and high-quality studies agree	Practice considerations
**Limited**	Medium (50–85%)	One high-quality OR Two medium-quality OR One medium-quality and one high-quality	If two studies (medium and/or high-quality) agree.<br><br>If more than two studies, more than 1/2 of the medium and high-quality studies agree	Not enough evidence from the scientific literature to guide current policies/practices
**Mixed**	Medium and high	Two	Findings from medium and high-quality studies are contradictory	Not enough evidence from the scientific literature to guide current policies/practices
**Insufficient**	No high-quality studies, only one medium-quality study, and/or any number of low-quality studies			Not enough evidence from the scientific literature to guide current policies/practices
